# Attitudes and behaviours of consumers towards plant‐based yoghurt alternatives: a cross‐cultural study

**DOI:** 10.1002/jsfa.70638

**Published:** 2026-05-19

**Authors:** Mitali K Gupta, Jeremy J Cottrell, Damir D Torrico, Melindee Hastie, Sally L Gras, Frank R Dunshea, Jing Lei

**Affiliations:** ^1^ CSIRO, Food Innovation Centre Werribee Australia; ^2^ School of Agriculture and Food, Faculty of Veterinary and Agricultural Sciences The University of Melbourne Parkville Australia; ^3^ College of Agricultural, Consumer and Environmental Sciences University of Illinois at Urbana‐Champaign Urbana IL USA; ^4^ Department of Chemical Engineering and The Bio21 Molecular Science and Biotechnology Institute The University of Melbourne Parkville Australia; ^5^ Faculty of Biological Sciences University of Leeds Leeds UK; ^6^ Faculty of Business and Economics The University of Melbourne Parkville Australia

**Keywords:** affective, dairy alternatives, cognitive, cross‐cultural, ethnic, social, vegan

## Abstract

**BACKGROUND:**

Increased awareness of sustainable food production and vegan, vegetarian and flexitarian diets are driving the growth in dairy substitutes, particularly plant‐based yoghurts. To understand consumer perception or mind‐set and facilitate adoption of plant‐based yoghurt products, a multi‐phase study using both qualitative and quantitative research methods was conducted to understand the differences between perceptions (cognitive, affective, and social responses) of consumers, belonging to Western and Asian cultural groups, to plant‐based yoghurts compared with traditional dairy yoghurts. The study involved a qualitative focus group (*n* = 37, four sessions), a quantitative survey (*n* = 433) and a quantitative blind‐tasting sensory experiment (*n* = 117), examining consumers perceptions and overall liking of the plant and dairy products. The focus groups produced terms used for further experiments.

**RESULTS:**

The quantitative survey showed that dairy yoghurts were preferred with higher overall liking scores compared to plant‐based yoghurt alternatives, and displaying differential cognitive, affective, and social responses. However, these perceptions contrasted with blind consumer tasting panel results, which showed that both types of yoghurts were similarly liked by respondents producing similar overall liking scores. Furthermore, the studies found that consumers' negative responses towards plant‐based yoghurt alternatives are primarily responsible for their lower popularity. Association towards emotion and health factors were common for both dairy and plant‐based yoghurts as shown by linear models. However, dairy yoghurts were additionally associated with ingredient and cultural factors, and plant‐based yoghurts were affected by social factors.

**CONCLUSION:**

Hence, addressing and overcoming the negative expectations regarding plant‐based yoghurts is essential to facilitate their future adoption. The study has important implications for novel product development strategies to facilitate consumer adoption of plant‐based yoghurts, especially for different cultural groups. © 2026 The Author(s). *Journal of the Science of Food and Agriculture* published by John Wiley & Sons Ltd on behalf of Society of Chemical Industry.

## INTRODUCTION

Dairy‐based yoghurts are popular products consumed globally in both drinkable and spoonable format. These products are a good source of probiotic bacteria, calcium, vitamins and minerals that can help to manage weight and assist with conditions such as irritable bowel syndrome (IBS).^1^ Recently, a number of factors have been driving the development of yoghurt products from alternative plant based sources, including the growing needs of the rising population, the need for greater sustainability and the increase of flexitarian diets.^2^, ^3^, ^4^ Plant‐based yoghurt alternatives have the potential to fulfil health and environmental needs if they can be acceptable to consumers in terms of taste and are a potential alternative protein source for vegan and lactose‐intolerant consumers.^5^ The consumption of plant‐based yoghurts can increase fibre, antioxidant, and polyphenol intake, depending upon the processing techniques and ingredients. However, market share is less than traditional dairy yoghurts.^6^ Given that market performance is typically driven by consumer liking,^7^, ^8^ this research examines the factors affecting overall consumer liking of plant‐based yoghurt in comparison with traditional dairy‐based yoghurt.

A combination of factors can affect the overall liking of a product, these include the inherent properties of texture and microstructure, the physicochemical attributes such as sensory liking (taste preference) and the cognitive, affective and social responses of consumers to such products.^9^, ^10^ Previous studies have shown that selected plant‐based yoghurts can have similar inherent properties to dairy yoghurts, leading to similar sensory liking.^11^ However, it is less clear whether consumers have different cognitive, affective, and social responses to plant‐based yoghurt compared to traditional dairy‐based yoghurt and whether these differences lead to the differential overall liking of these yoghurt products. Therefore, we examined the cognitive responses by assessing consumers' perceived health benefits and ingredient expectations for each yoghurt product type. In addition, we examined emotional responses by assessing the emotions that consumers linked to these yoghurt products and social responses by measuring the perceived impact of each yoghurt on diet and sustainability.

Furthermore, it is well‐acknowledged that consumers are not a homogenous group, with consumer responses and preferences for the same food product varying depending on cultural background.^6^ For instance, in yoghurt consumption, consumers from Western countries tend to consume spoonable sweetened yoghurts, whereas consumers from Asian countries are more familiar with drinkable yoghurt products or use yoghurt as a savory ingredient.^12^ Therefore, we also examined how the consumer response to the two types of yoghurt formats differed between the two different cultural groups, Western and Asian consumers. The key objective of this research was to examine consumers' cognitive, affective, and social responses to plant‐based yoghurts compared to dairy‐based yoghurts and to assess the impact of these responses on overall liking and consumer acceptance of plant‐based yoghurt products.

## METHODOLOGY

A mixed‐method approach was used in the study, which included:

1. a focus group study to explore the cognitive responses (perceived health benefits and ingredient expectations), affective responses (related to emotions) and social responses (related to diet and sustainability) of consumers to dairy and plant‐based yoghurt products for the two broad cultural groups, Western and Asian consumers.
2. a survey study to measure and quantify the impact of cognitive, affective, and social responses on overall consumer liking of dairy and plant‐based yoghurt products.
3. a blind‐tasting sensory study to compare the actual liking during taste test between the dairy and plant‐based yoghurt products.

All study experiments were approved as a low‐risk investigation by the Human Ethics Committee of FVAS (Ethics ID 1853507.2) at The University of Melbourne.

### Study 1

#### Qualitative study for cognitive and social responses

A facilitated focus group approach was used for the qualitative study to understand the differences in cognitive health benefits and ingredient expectations regarding dairy yoghurts and their plant‐based alternatives. This study aimed to identify the most selected terms for each yoghurt category and to determine any differences between the two cultural categories. Participants were recruited through emails and newsletters and signed a consent form before participation.

##### Participants and facilitated session

Untrained consumers (*n* = 37) were recruited for the focus group study, held in four different sessions, where participants were divided into cultural groups by self‐identifying themselves (two focus groups for each culture): Asian (*n* = 18) and Western (*n* = 19). A brief introductory discussion was used to encourage participant engagement. The sessions for each cultural group were held separately, so that interpretation of the result is uniform. This session followed a semi‐structured discussion format commonly used in qualitative consumer research. All sessions (1 h each) were led by a facilitator, where the participants were told about the different product types (dairy and plant‐based yoghurts) to be discussed in the session. Representative images of the different yoghurt types were displayed to the participants on the table.^13^ Further, a descriptor mapping exercise (as mentioned in the next section ‘Perceptual mapping’ section) was carried out, and finally, a general discussion about the consumption and preference of yoghurts took place. No tasting of yoghurt products was conducted during the focus group sessions.

##### Perceptual mapping

###### Cognitive and social responses

**Figure 1:**
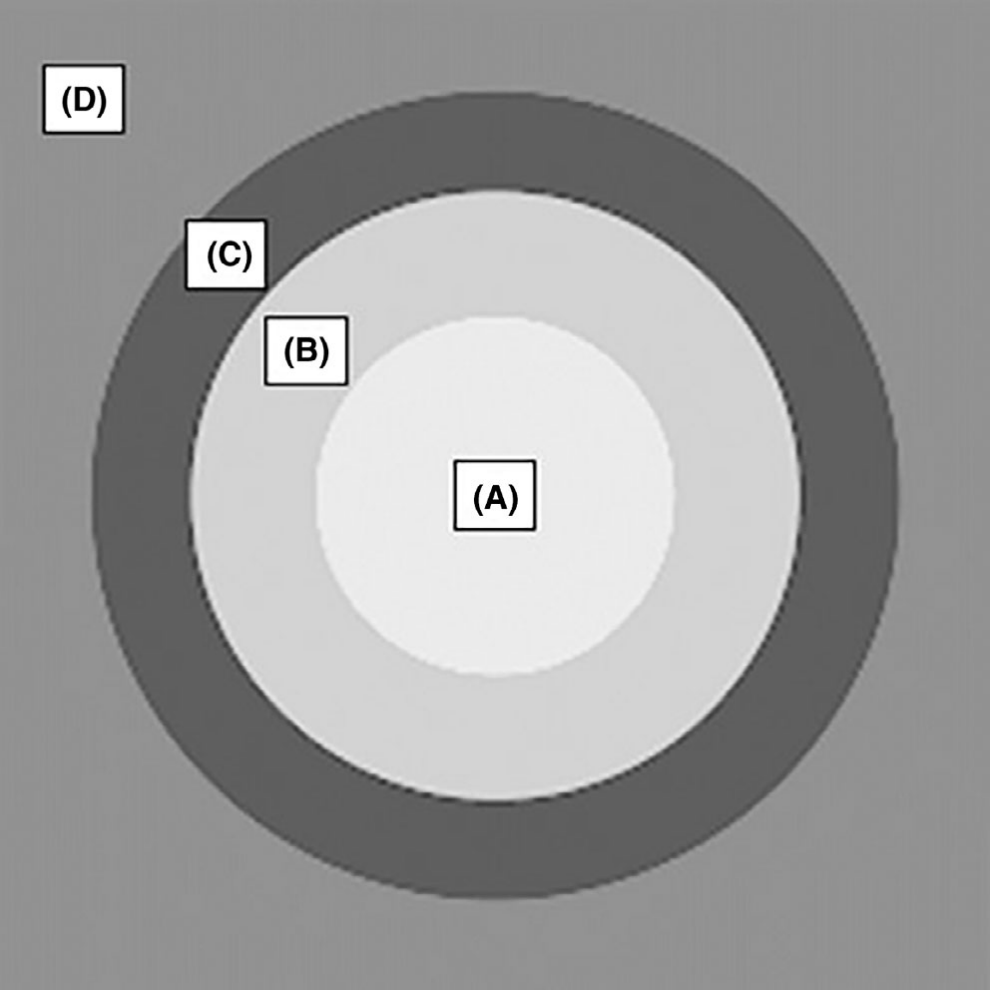
Depicts how the circles were placed where participants were asked to identify the words according to relevance, (A) high priority region, (B) medium priority region, (C) low priority region, and (D) not relevant.

Perceptual mapping was carried out to understand consumer responses to the two product categories and shortlist the terms for perceived health benefits, ingredient expectations and social benefits for further quantitative study. Participants were randomly split into two sub‐groups and each group was asked to answer the differences between dairy and plant‐based yoghurt consumption habits. Each group was separately asked to discuss and place the words from their knowledge, experience, or perception on the chart, marked with three concentric circles in order of relevance (as depicted in Fig. 1). The order of priority included region A (most relevant or highest priority descriptors), region B (medium relevance or medium priority descriptors), region C (low relevance or low priority descriptors) and region D (not relevant descriptors). Participants first started with dairy yoghurts and continued with plant‐based yoghurts. Instead of using the *x–y* plot approach for perceptual mapping followed by Lopetcharat and Beckley,^14^ the study was modified by using concentric circles. Participants were asked to place the terms for dairy yoghurts onto the concentric‐circle chart based on perceived relevance. The order of preference referred to the priority regions on the chart, where placement in region A indicated the highest priority and region D indicated no relevance, rather than a numerical or sequential ranking of terms. The use of concentric circles for perceptual mapping is a recognized qualitative visual elicitation approach that allows participants to prioritize concepts based on perceived relevance rather than forcing placement along predefined dimensions. This method is particularly suitable for exploratory consumer research, where the objective is to identify salient attributes and beliefs that inform subsequent quantitative testing. Compared with traditional *x–y* perceptual maps, concentric circle mapping reduces cognitive load and facilitates group discussion, enabling participants to draw on prior knowledge and experience when ranking product‐related attributes.^15^

Further, lists (List 1 and List 2, Table 1) of perceived health benefits, ingredient expectations and terms of social benefit were provided to the participants (each list provided to each group), which they were then asked to place in the order of preference for dairy yoghurts, as explained previously. These lists were based on a combination of health, ingredient and social benefit terms and were compiled from previous literature based on benefits generally offered by commercial yoghurt products.^16^, ^17^, ^18^ List 1 included all the primary benefits of yoghurt and List 2 included advanced benefits. The same exercise was repeated for plant‐based yoghurts, by exchanging the lists between the two groups.

**Table 1:** List of words given to participants that they would relate to dairy and plant‐based yoghurts

List 1	List 2
Low fat	Extra protein
Low sugar	Additional antioxidants
Easy to digest	Extra calcium
Quick and easy snack	Full of probiotics
Fills me up	Full of phytochemicals
Helps to manage weight	Builds immune system
Steady supply of protein	Healthy heart
Deals with my hunger cravings	Helps repair muscle (post work‐out)
GI (glycemic index) management	Lean muscle development
Healthy and nutritious	Better sleep
Fits my daily routine	Glowing skin
Vitamins and minerals	Omega‐3 fatty acids
Prevents osteoporosis	Promotes better mood
Prevents irritable bowel disease	Eat with antibiotics (for healthy tummy)
Full of healthy dairy	Sustainability (environmental)
Vegan	Animal welfare

###### Affective responses

The emotion terms were selected on a previous focus group study with consumers (*n* = 32),^10^ reflecting a combination of positive, negative, and neutral emotional responses. (Supporting Information, Table S3).

### Study 2

#### Quantitative survey study

The consumer survey was created to measure the acceptance of the dairy and plant‐based yoghurt types, understand the cognitive, affective, and social responses of consumers, and further quantify the relative impact of different responses on overall consumer liking of different yoghurt products.

##### Development of online survey

Using the online Qualtrics XM (Qualtrics, Provo, UT, USA) platform (2019), the survey was designed on the following four main concepts: (1) the overall liking of the two types of yoghurt (dairy and plant‐based) by consumers and their consumption patterns of these yoghurts, (2) the cognitive (perceived health benefits and ingredient expectations), affective (emotions) and social (diet and sustainability) responses of consumers to the two types of products, and (3) consumer demographics including their cultural background and their social and perceived values of the two types of yoghurts.

In the survey, participants were first asked in parallel to rate their overall liking of the dairy and plant‐based yoghurt on the nine‐point hedonic scale (1 – dislike extremely, 5 – neither dislike nor like, 9 – like extremely).

Next, participants were asked to select all the terms they related to health benefits, ingredient expectations, emotions, and social benefits for both dairy and plant‐based yoghurts from the list of terms provided. These terms were previously selected in the focus group studies, following the check‐all‐that‐apply (CATA) method.

Participants were also asked to indicate if they have tried a plant‐based yoghurt alternative previously, whether they preferred coconut, soy, almond or other (rice, oat) products. Willingness to pay for the plant‐based yoghurts was also recorded as compared to the dairy yoghurts, in terms of ‘more’, ‘less’, ‘same’ or ‘won’t buy’.

In addition, respondents were asked for their preferred time of consumption of yoghurts.

##### Participants

A total of *n =* 433 respondents were recruited through The University of Melbourne online portals, newsletters, alumni network, and social groups. The survey was taken by respondents, with demographics (Table S1) divided into: gender – female and males; age group – 17 to 24 years, 25 to 34 years, 35 to 44 years and above 44 years; ethnicity – Westerners and Asians; work status – work full‐time, work part‐time and student; food consumption habits – not on a specific diet, flexitarian, vegan/vegetarian and others, which include gluten or dairy free, low glycemic index (GI) diet, keto diet and pescatarian diets.

### Study 3

#### Sensory tasting study

In addition to the quantitative survey, another objective of the study was to compare whether actual experience would contrast or align with consumer perceptions. Hence, a blind tasting study with two plain test yoghurts (a dairy and a plant‐based yoghurt) was carried out to compare consumer liking and affective responses in actual tastings, with the consumer responses measured in the quantitative perception survey study. This comparison was based only on affective responses and taste perception, as it was assumed that taste, not cognitive or social perceptions, would be the primary determinant of liking scores and emotions.

##### Participants

One hundred and seventeen student and staff members at The University of Melbourne participated in the blind‐tasting study in individual sensory booths. Participants were asked to taste yoghurts and rate overall liking on a scale of 1–9 (where, 1 – dislike extremely, 5 – neither dislike nor like, 9 – like extremely) and to also select all emotion terms (from a list of terms provided using CATA; see ‘Development of online survey’ section) that related to each of the tasted yoghurts. The emotions were measured to understand how consumers related these during the blind tastings. The range of the demographic details of the participants were: age: 20–68 years; gender: 42 males and 75 females; ethnicity: 73 Asian and 44 Western.

##### Samples and tasting

The two test plain yoghurts were commercial products selected to avoid any influence of sugar on consumer ratings. These were selected by a focus group study conducted previously.^10^ The samples were labeled with a three‐digit code and were served in a random order in 15 mL cups. The data was collected using the Samsung Galaxy View 18″ tablet (Seoul, South Korea) within the Bio‐Sensory app (The University of Melbourne, Melbourne, Australia). The temperature of the room was maintained at 22–24 °C and the serving temperature of the yoghurt was 8–10 °C. There was at least a 1‐min break between tasting two individual samples and participants used water and crackers (Captain's Table, classic water crackers; purchased in Melbourne, Australia) as palate cleansers.

### Data analysis

In the qualitative study 1, the research team analysed the session notes and insights were generated for the two yoghurt categories. The terms were grouped into high, medium, low and no priority sections for each of the two yoghurt types, based on Asian and Western cultures. In the quantitative studies 2 and 3, Minitab software (version 19.1; Minitab Inc., State College, PA, USA) was used to measure the mean overall liking scores and sustainability scores. The differences were estimated by one‐way analysis of variance using Ficher's least significant difference (LSD) comparison test, with a significance level of *P* < 0.05. The interaction of overall liking with demographics was measured for the survey participants. A general linear model was established to link the emotions, health benefits and ingredient expectations with an overall liking score (as fixed factors) for dairy yoghurts and their plant‐based substitutes, with culture as a co‐factor. The CATA analysis and principal coordinate analysis (PCoA) for the cognitive and affective responses, as ranked by participants, was performed using XLSTAT (version 2020; Addinsoft, New York, USA). Cochran's *Q* test (at *P* ≤ 0.05) was used to compare for ratings given by Western and Asian groups on CATA data.

## RESULTS

### Study 1

#### Perceptual mapping results in cognitive and social responses

Table 2 shows the cognitive responses as rated by consumers, in order of relevance, for both the dairy and plant‐based yoghurt product categories. The health benefits of ‘bone health’ and ‘digestion’ were rated as the highest priority for dairy, whereas ‘healthy heart’ was rated as the highest priority for plant‐based yoghurt. Cognitive terms like ‘better sleep’ and ‘glowing skin’ showed no priority for either product category. Ingredient terms including ‘low sugar’ showed the highest priority for both the products, whereas ‘phytochemicals’ showed no priority for dairy but high priority for plant‐based yoghurt. Other medium and low relevance terms are also listed in Table 2. The social terms ‘environmental’ and ‘sustainability’ showed the highest priority for plant‐based yoghurts, whereas no priority was attributed to these terms for the dairy product. The qualitative study could not distinguish well between the attributes related to each ethnic group. However, this helped to understand the relevant attributes (i.e., the most chosen terms), which were further divided into cognitive benefits (health benefits and ingredient expectation) and social benefits (sustainability and diet) and these categories were then used in the behavioural survey to quantify these parameters.

**Table 2:** Cognitive attributes as rated by Western and Asian consumers in order of relevance

Group	Type	Highest priority	Medium priority	Low priority	No priority
Dairy	Health benefits	Bone health	Manage weight	Builds immune system	Better sleep
		Digestion	Gut health	Healthy heart	Glowing skin
			Prevents osteoporosis	Prevents irritable bowel disease	
	Ingredient expectation	Calcium	Low fat		Phytochemicals
		Probiotic	Vitamins and minerals		
		Extra protein			
		Low sugar			
	Sustainability and diet			Easy cultivation	Environmental sustainability
					Animal free
Plant	Health benefits	Healthy heart	Builds immune system	Muscle repair	Better sleep
					Prevents irritable bowel disease
					Glowing skin
	Ingredient expectation	Full of probiotics	Steady supply of protein		
		Omega 3 fatty acids	Extra protein		
		Phytochemicals	More fibre		
		Low sugar	Additional antioxidants		
	Sustainability and diet	Environmental sustainability	Food for future	Water conservation	
		Vegan	Ethical		

### Study 2

#### Online survey results

Consumers perceived dairy and plant‐based yoghurts differently and gave a higher overall liking score to the dairy products (7.21 of 9) in the online survey, as shown in Table 3.

**Table 3:** Comparison of mean overall liking scores for a dairy yoghurt with a plant‐based yoghurt, as perceived by respondents (overall liking on a scale of 1 to 9)

Product	Overall liking
Dairy	7.21 ± 1.90^a^
Plant	5.19 ± 1.71^b^

*Note:* Means with different superscript lowercase letters in each column indicate significant differences (*P* < 0.05) by the Fischer's least significant difference (LSD) test.

##### Relationship between the demographic factors and overall liking of yoghurt products

**Figure 2:**
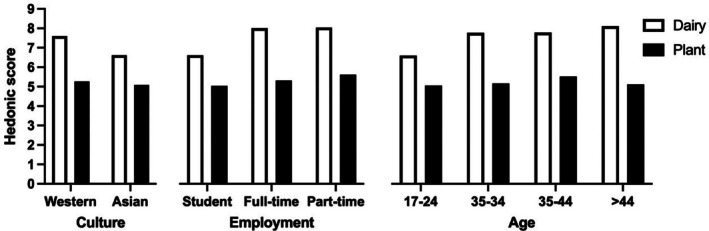
Comparison of the mean overall liking scores for dairy yoghurt and plant‐based yoghurt substitutes, according to demographic factors.

The demographic factors of consumers, including age, ethnicity (culture) and work status influenced the overall liking ratings for dairy and plant‐based yoghurts (*P* < 0.05). There were no significant effects detected for consumption habits and gender (*P* > 0.05). Figure 2 displays the average overall liking scores for dairy yoghurts and plant‐based yoghurt substitutes, according to the demographic factors. Table S2 further explains in detail the interaction plot for liking with the demographic factors.

*Age group* – The younger group, aged 17–24 years, had a lower ranking for dairy liking (score 6.60) compared to other age groups (scores 7.77, 7.78 and 8.12 for 25–35 years, 35–44 years and > 44 years, respectively). The liking of plant‐based yoghurt was ranked lower by all age categories (scores 5.06, 5.17, 5.52 and 5.12 for 17–24 years, 25–35 years, 35–44 years and > 44 years, respectively) and was not significantly different between the age groups.

*Culture* – Participants from a Western country gave a higher liking score to dairy (score 7.61), compared to participants from an Asian country (score 6.62), whereas participants from both cultures ranked plant‐based yoghurt (score 5.27 by Westerners, score 5.09 by Asians) equally, which was lower than dairy.

*Based on work status* – Plant‐based yoghurt was less liked by all consumer categories (scores 5.62, 5.31 and 5.04 for working part‐time, working full‐time and students, respectively) as compared to dairy yoghurt (scores 8.04, 8.01 and 6.62 for working part‐time, working full‐time and students, respectively), with the student group having a lower liking of yoghurt (both dairy and plant) compared to participants who were working full‐time or part‐time.

*Food consumption habits* – The interaction of food consumption habits with the product type was not significantly different (*P* > 0.05; results not reported). This is probably due to a low sample size for vegans/vegetarians, which was not sufficient to make any conclusions.

*Gender* – The interaction between product and gender was not found to be significant (*P* > 0.05). Hence, participants who identified as female or male did not show any difference in the liking of dairy yoghurts and plant‐based alternatives based on gender.

Culture is one of the most important demographic factor affecting the overall liking of yoghurt products, hence this factor has been explained in detail in further sections.

##### Cognitive, affective, and social responses related to the consumption of yoghurts: check‐all‐that‐apply (CATA) methodology

Participants were asked to select all terms they would link with the cognitive factors, including health benefits and ingredient expectations, as well as affective factors, including emotions, and their cultural comparisons are presented in Table 4.

**Table 4:** Proportions (out of one) as rated by participants, describing dairy yoghurts and their plant‐based alternatives for ingredients

Groups	Terms	Western consumers		Asian consumers	
		Dairy	Plant	Dairy	Plant
*Health benefits*					
Bones	Prevents osteoporosis	***0.496***	0.092*	***0.254***	0.110 #
	Stronger bones	***0.696***	0.123*	***0.503***	***0.237*** #
Gut	Improves gut health	***0.742***	0.377*	***0.543***	0.329 #
	Helps digestion	0.385	0.300*	0.428	0.324 #
	Prevents irritable bowel syndrome	0.181	***0.104*** *	0.214	***0.179***
General health	Helps repair muscle	0.277	0.112*	0.22	0.116 #
	Manage weight	0.265	***0.269***	0.353	***0.434***
	Builds immune system	0.312	***0.162*** *	0.393	***0.306***
Well‐being	Healthy heart	0.131	***0.165***	0.191	***0.254***
	Better sleep	***0.054***	***0.065***	***0.15***	***0.202***
	Glowing skin	***0.065***	***0.112*** *	***0.208***	***0.283*** #
*Ingredient expectation*					
Basic benefits	Low fat	0.246	0.119*	0.329	0.162 #
	Low sugar	***0.496***	0.115*	***0.387***	0.139 #
	Extra protein	0.485	0.062*	0.399	0.104 #
	Extra calcium	***0.723***	0.023*	***0.497***	0.052 #
	Full of probiotics	***0.681***	0.096	***0.486***	0.104 #
Advanced benefits	Vitamins and minerals	0.262	0.108*	0.358	0.162 #
	Omega‐3 fatty acids	0.069	0.023*	0.127	0.040 #
	Full of phytochemicals	***0.008***	0.035*	***0.052***	0.052
	Additional antioxidants	0.081	0.069	0.127	0.098
	More fibre	0.046	***0.062***	0.069	***0.162*** #
*Emotions*					
Positive	Cheerful	0.331	0.127*	0.353	0.185 #
	Trusted	0.469	***0.104*** *	0.393	***0.179*** #
	Uplifting	0.162	0.092*	0.162	0.116
	Luxury	0.085	0.212*	0.087	0.231 #
	Dependable	***0.431***	0.038*	***0.266***	0.075 #
Negative	Deceitful	0.015	0.038	0.017	0.029
	Artificial	0.023	0.208*	0.035	0.156 #
	Pretentious	0.004	***0.238*** *	0	***0.075*** #
	Nasty	0.023	0.05	0.012	0.035
	Cheap	***0.146***	0.023*	***0.231***	0.035 #
Neutral	Indifferent	0.127	0.142	0.092	0.179 #
	Neutral	***0.173***	0.165	***0.324***	0.220 #
	Guilt‐free	0.208	0.265	0.173	0.295 #
	Basic	0.042	0.046	0.029	0.081 #
	Common	0.323	0.019*	0.405	0.046 #
*Social benefits*					
Sustainability	Low carbon emission	***0.012***	0.150*	***0.121***	0.208 #
	Easy cultivation	0.123	***0.038*** *	0.168	***0.116***
	Water conservation	0.023	0.081*	0.029	0.046
Diet	Food for future	***0.050***	0.231*	***0.116***	0.312 #
	Vegan	***0.008***	0.688*	***0.052***	0.630 #
	Ethical	0.054	0.331*	0.081	0.353 #
	Animal free	***0.012***	***0.665*** *	***0.052***	***0.491*** #

*Note:* * and # indicating significant difference between samples (dairy and plant) according to Cochran's *Q* test at *P* ≤ 0.05 in Western and Asian cultural groups, respectively. (Cochran's *Q* test carried out independently for each of the cultures). The values in bold are significantly different across cultures according to Fisher's exact test.

For the overall health benefits, consumers had a higher expectation of bone and gut health for dairy products. In contrast, consumers rated the well‐being group higher for plants, with the exception of ‘better sleep’, which was rated equally for both products. A similar trend was observed across cultures. However, Western participants rated ‘prevents osteoporosis’, ‘stronger bones’ and ‘improves gut health’ to be higher for dairy, whereas Asian consumers rated ‘glowing skin’ and ‘better sleep’ to be higher for dairy. In the case of plant‐based yoghurts, ‘prevents IBS’, ‘stronger bones’, ‘manages weight’, ‘builds immune system’, ‘healthy heart’, ‘better sleep’ and ‘glowing skin’ were rated higher by Asian consumers as compared to Western consumers.

In the case of ingredients, consumer expectations were higher for basic and advanced benefits groups for dairy, with the exception of ‘full of phytochemicals’ and ‘additional antioxidants’, which were similar for both products, and plant yoghurts rated higher for ‘more fibre’ (Table 4). Again, these trends were similar across cultures. However, Western consumers considered dairy yoghurts to be ‘full of probiotics’, ‘low sugar’ and ‘extra calcium’, compared to Asian consumers. Asian participants also felt plant‐based yoghurts contained ‘more fibre’ than the Western consumer group.

Comparing affective responses in terms of the associated emotions for the two product categories, dairy yoghurts were associated more with positive terms, except for the term ‘luxury’. The plant‐based yoghurts were highly associated with negative terms, except for the term ‘cheap’. A similar trend was observed across cultures, as for other trends observed earlier. However, the dairy yoghurt was rated to more ‘dependable’ by Western consumers, while Asian consumers rated these products as ‘cheap’ and ‘neutral’. The plant product was also considered to be more ‘pretentious’ by the Western consumer group, whereas it was viewed as more ‘trusted’ by Asian consumers.

For social benefits, plant‐based yoghurts received higher sustainability scores than dairy yoghurts. This sustainability focus was notable for Western consumers, who rated plant‐based yoghurts high for sustainability group terms (Table 4), while these were rated equally Asian consumers, except for ‘ low carbon emission’. Both cultural groups rated diet terms higher for plant‐based yoghurts than dairy yoghurts.

##### Model to link dairy and plant‐based yoghurts with the cognitive and affective responses and overall liking

A linear model was developed separately for dairy and plant‐based yoghurts to relate the health benefits, ingredient expectations, emotion terms, social benefits, and culture, with the overall liking as the response variable. The general linear model explained 31.5% and 18.4% variance for the dairy‐ and plant‐based yoghurt groups, respectively. The selected terms (significant; *P* < 0.05) are shown in Table 5.

**Table 5:** Top descriptors related to overall liking scores in the linear model

Factors	Variables	Difference of levels	Difference of means	Individual 95% confidence interval	*P*‐Value	Percentage variance explained
*Dairy‐based*						
Emotions	Dependable	1–0	1.18	(0.70, 1.65)	<0.001	31.5%
	Neutral	1–0	1.16	(0.70, 1.62)	<0.001	
	Nasty	1–0	−2.51	(−3.49, −1.52)	<0.001	
Health	Gut health	1–0	0.70	(0.29, 1.10)	<0.001	
Ingredient	Phytochemicals	1–0	−1.34	(−2.32, −0.36)	0.007	
Culture	Western, Asian	Western–Asian	−1.33	(−1.66, −1.00)	<0.001	
*Plant‐based*						
Emotions	Cheerful	1–0	0.64	(0.22, −1.07)	0.003	18.4%
	Luxury	1–0	0.49	(0.13, 0.85)	0.008	
	Nasty	1–0	−3.51	(−5.20, −1.83)	<0.001	
	Deceitful	1–0	0.89	(0.28, 1.51)	0.002	
	Guilt‐free	1–0	0.45	(0.11, 0.79)	0.010	
Health	Gut health	1–0	0.38	(0.07, 0.69)	0.018	
Social	Animal free	1–0	0.43	(0.120, 0.736)	0.007	

For the dairy yoghurt, the overall liking was positively correlated to the emotion terms ‘dependable’ and ‘neutral’, increasing the mean liking score by 1.18 and 1.16, respectively. In contrast, overall liking was negatively correlated to ‘nasty’, which decreased the mean liking score by 2.51. ‘Gut health’ increased the mean overall liking score by 0.70; however, ‘phytochemicals’ reduced the mean liking score by 1.34. Furthermore, the difference in culture for Western consumers and Asian consumers affected the mean score by 1.33, resulting in a higher value for the Western consumer group. Social responses did not affect the mean for dairy‐based factors.

In the case of the plant‐based yoghurt, the liking was positively related to the emotion terms ‘cheerful’, ‘luxury’ and ‘guilt‐free’, increasing the mean scores by 0.61, 0.49 and 0.45, respectively. In contrast, liking was negatively linked to the terms ‘nasty’ and ‘deceitful’, decreasing the mean score by 3.51 and 0.89, respectively. The liking score increased by 0.38 for ‘gut health’ and increased by 0.43 for ‘animal free’. However, the effect of culture and ingredient was not significant for plant‐based yoghurt products.

##### The social and perceived value of dairy yoghurts and their plant‐based alternatives

**Figure 3:**
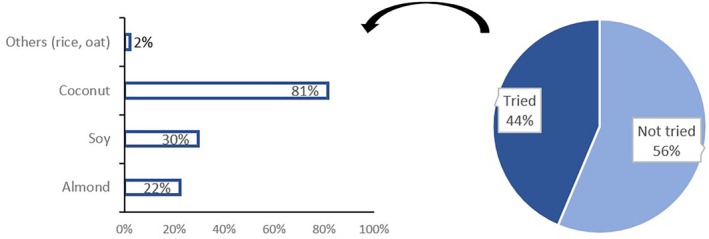
Respondents who have tried plant‐based yoghurts (44%) and liking for the different plant‐based yoghurt options.

**Figure 4:**
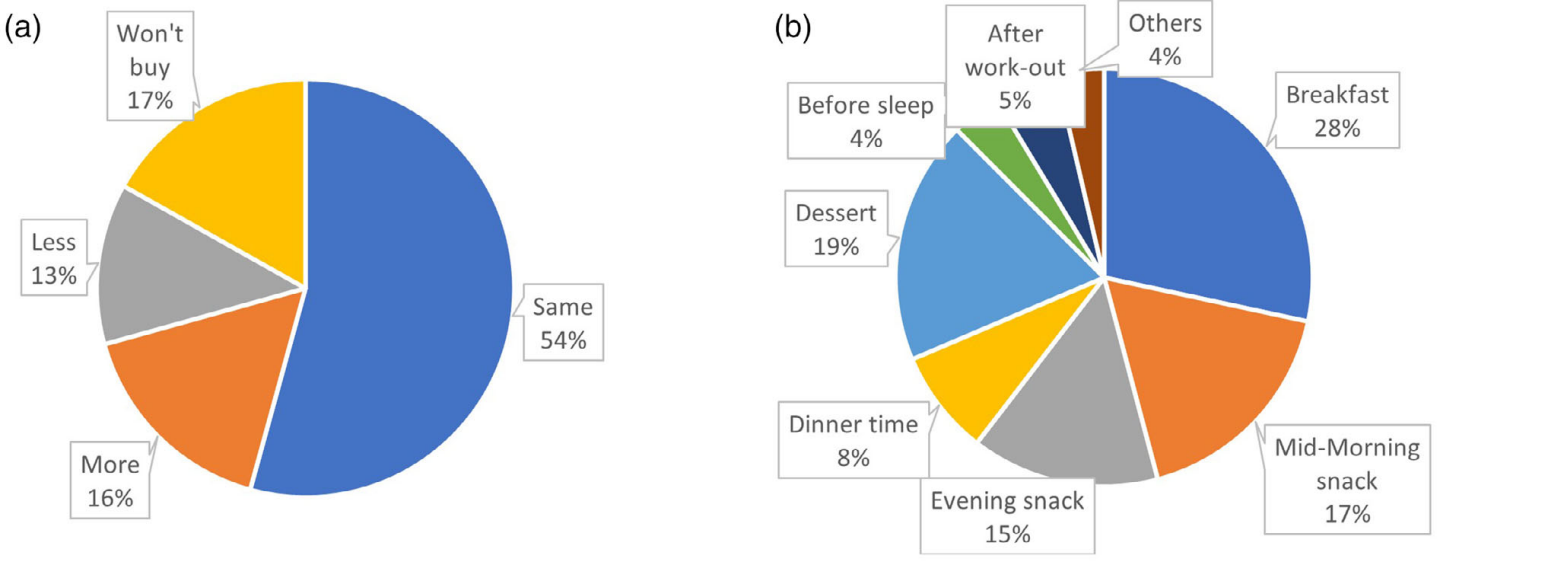
Willingness to pay for plant‐based yoghurt alternatives compared to dairy (a), and preferred time of consumption for the dairy yoghurts (b).

A total of 44% of respondents in the survey had previously tried a plant‐based yoghurt alternative. Among those participants, 81% rated coconut yoghurt as the most liked and popular type, followed by 30% for soy and 22% for almond (Fig. 3). A total of 54% of respondents were willing to pay the same price as their regular dairy yoghurts, whereas 16% were willing to pay even higher than the regular price. A total of 13% of participants would prefer to pay less, and the remaining 17% would not be interested in purchasing such yoghurts (Fig. 4(a)). Out of *n* = 433, 28% prefer to consume yoghurt for breakfast, 19% as dessert, 17% as a mid‐morning snack, 15% as an evening snack and the rest 21% in dinner time, before sleep and after a workout (Fig. 4(b)). Understanding consumers and their interests is important for developing a product development strategy.

### Study 3

#### Comparing yoghurt perception with actual tasting

**Figure 5:**
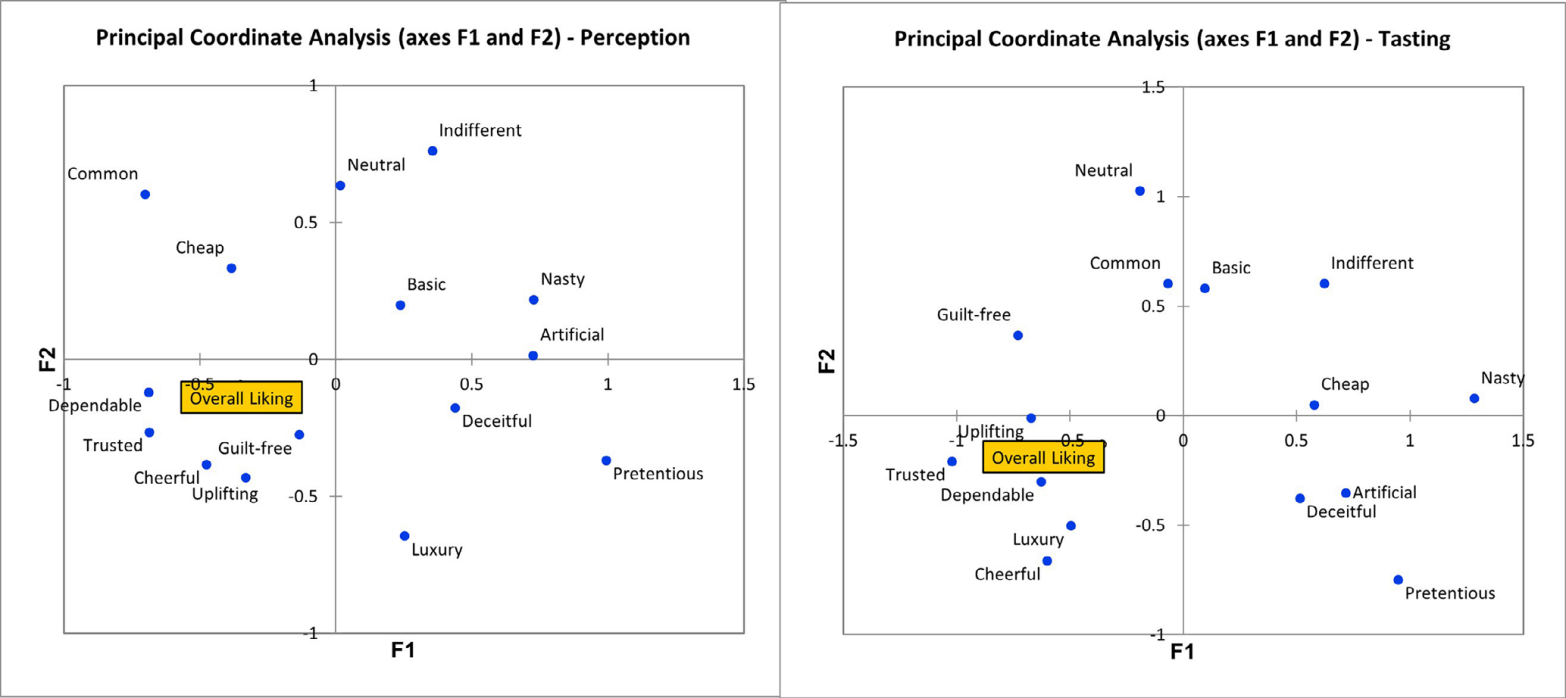
Comparison of principal coordinate analysis for the relation of overall liking with the emotion terms for perception‐based and actual tasting‐based analysis.

As observed in the previous survey, study 2, plant‐based yoghurts are perceived as less healthy and linked with more negative emotions. A further blind‐tasting study was conducted to understand the actual differences between consumer perception and actual tasting experience. A range of yoghurts, including plain dairy and soy (plant‐based) were tasted by consumers (*n* = 117) and overall liking was rated. Both samples ranked similarly in the blind tasting experiment, with an overall liking score of 5.35 (out of nine). However, in the survey study of yoghurt perception, which did not involve any tastings, the consumers overall ranked plant‐based yoghurt (5.19) much lower than dairy yoghurt (7.21). This difference was consistent for both the cultural groups. However, the overall liking for both products was linked to similar emotion (positive) terms in both the perception and tasting studies, as observed in the PCoA (Fig. 5).

## DISCUSSION

Consumers are the final step in the production chain; hence it is helpful to identify factors that affect their behaviour and product liking. Products must be consumer‐oriented to fulfil the cognitive and affective needs of the consumers.^19^ This includes social aspects (environment and sustainability). Consumers are also affected by many external factors including culture, product awareness^20^ and motivation.^21^ Culture can affect acceptance to inherent properties and familiarity with the ingredients. Product awareness and motivation can affect the marketability of yoghurts. Affection can influence the liking and dissatisfaction can arise due to negative affection.^22^

The multi‐phase approach in this study has helped in understanding the different factors that can influence consumer liking. The focus group suggested that consumers are seeking similar health benefits from yoghurt products and expect similar ingredient benefits from both product categories. This further suggests that the consumers are more concerned about direct and individualistic benefits, rather than indirect or abstract properties, such as the welfare of animals or the environment where these yoghurts are produced. The qualitative sensory study did not differentiate between the two cultures but helped to select the terms for further quantitative sensory.

### Why does the difference exist?

Perception‐based data from the online survey showed differences in consumer liking scores for the two yoghurt categories, with traditional dairy yoghurts rating higher than plant‐based yoghurts. These variations can arise due to a combination of cognitive, affective, and social factors towards these novel plant‐based yoghurts. The differences can also be attributed to the cultural background of consumers, which is the most important demographic factor affecting ingredient familiarity, as validated in another study of cultural influence towards food liking.^23^ Other factors affecting liking include demographic factors such as age and work status.

#### Demographic differences

An understanding of consumer likings and consumption habits can assist product development and marketing. In study 2, the ‘Students’ group rated plant‐based yoghurts lower than dairy yoghurts. This could be attributed to the lower awareness of the taste and health benefits of plant‐based products. Wu *et al*.^24^ similarly compared the acceptability of soy‐based yoghurts with dairy yoghurts and found that college students were unaware of the health benefits of soy; hence, they preferred traditional dairy products. The researchers in this study recommended that nutritional advice could increase understanding and product uptake.

Younger consumers (aged 17–24 years) ranked dairy yoghurts lowest in liking scores compared to other consumer groups aged > 25 years. This difference could be attributed to a negative attitude towards dairy, as younger consumers can consider such products to be less healthy.^25^ In another study by Johansen *et al*.,^26^ the important motives for choosing reduced‐calorie yoghurts were studied. They found that younger consumers were mainly motivated by low‐fat content, weight control and good taste across several countries. Whereas healthiness was an important motive for food choice for older consumers. Younger consumers look for lower‐calorie yoghurt types and consider traditional yoghurts to be less healthy. In another study, 28% of consumers like to have yoghurt at breakfast, 32% have yoghurt as a mid‐morning or evening snack and 19% preferred yoghurt as dessert, which is consistent with the observations made here. In a study carried out in the European Union (EU), the majority of the consumers chose yoghurt as a dessert or as a snack.^27^ Results from the present and previous studies can help understand consumer preferences, based on various demographic variables, and can further help to build new product development strategies that target specific consumer groups.

The most important demographic factor influencing yoghurt consumption is culture. The present study found that both cultural groups, Western and Asian, prioritized different ingredients with their traditional yoghurts and related different emotional perceptions with the same yoghurt. A study comparing Chinese and New Zealand European consumer groups has shown a difference in consumer preference and yoghurt choice.^28^ This indicates that cultural background is an important factor that should be considered for developing yoghurt products that target specific markets.

#### Cognitive and affective differences

The consumer acceptability of yoghurts is affected by a variation in sensory attributes and emotions.^29^ It was observed that a major resistance to the success of a novel product in the market can be overcome by understanding the consumer psychological needs.^30^ As may be expected, positive emotions^31^ increased mean overall liking scores compared to negative emotions in a previous study^29^ and in a study where positive consumers emotions were related to well‐being and products high in fat and sugar were considered harmful.^32^ In this study, the Asian consumer group associated the plant‐based yoghurts with more positive emotions and health‐based terms compared to the Western consumer group, who perceived dairy yoghurts to be preferable, as discussed in ‘Cognitive, affective, and social responses related to the consumption of yoghurts: Check‐all‐that‐apply (CATA) methodology’ section. This behaviour could be because Asian consumers are culturally more familiar with plant‐based products, leading to positive cognitive and affective responses towards such product types. A similar trend occurred in a survey study for plant‐based meats in Asian countries,^33^ where cultural background likely also influenced product perception. A recent article also suggests that cross‐cultural variation in emotion concepts and attribute associations can meaningfully influence yoghurt acceptance even when hedonic liking is comparable between groups.^34^

#### Awareness and social benefits

The plant‐based yoghurts gained a higher sustainability rating than dairy yoghurts, even though these were perceived to be less liked in terms of taste. This showed that consumers need to be made aware of the extrinsic benefits of the product to increase popularity. Previous studies have shown that plant‐based yoghurts can be perceived as sustainable. A synergy between sustainability and health was studied, where average conscious consumers perceived sustainable products to be healthier than regular products.^35^ In another study for brand positioning of green restaurants, consumers showed an awareness towards green attributes and linked environmental sustainability, ingredients, and health benefits with a happier lifestyle. This association provides the opportunity for increased awareness amongst consumers and better positioning in the marketplace.^36^ It has been studied that eating motives are important in changing towards more sustainable food consumption patterns and plant‐based food consumption, which includes social image, as seen for meat/beef replacement.^37^

### Perceptions *versus* actual tastings

A blind sensory tasting experiment was conducted to understand liking and emotions towards the tasted samples. Similar liking scores were rated for both dairy and plant‐based (soy) samples. The study compared perceptual differences between the two yoghurt types using an online survey, where plant‐based yoghurt alternatives were reported to be less liked than dairy yoghurts. A commercial study showed a similar trend for liking perception.^38^ Comparable results were observed for another study where dairy and soy yoghurts were also equally liked on tasting.^39^ Another study further confirmed that both yoghurt types can be similarly liked in terms of their mouthfeel.^40^ It can be inferred that if a plant‐based yoghurt is created with an acceptable taste and texture, it can be made popular among consumers.^5^ This comparison showed that taste and texture are important factors for accepting of yoghurt products, but product knowledge can also affect consumer decisions. If consumers are not aware of the benefits and relevance of these products, this typically prevents purchase. Factors such as demographics and emotions can affect perception or acceptance towards a new product category.

### How to overcome the difference, and make such products more popular in the market?

Successful product design considers consumer attitudes and preferences. It is important to know when consumers like to consume yoghurt to formulate a product accordingly. In a study by Elzerman *et al*.,^41^ the use of meat substitutes was related to user intent, which is a critical factor in the product development strategy and a similar principle applies to yoghurts. Here, the perceptual properties of yoghurts were evaluated using a linear model (Table 5; study 2), which further helped in understanding the relationships between factors affecting liking for dairy and plant‐based yoghurt products. A combination of cognitive, affective, and social responses affected these products differently, a finding which can be addressed within product development strategies. Overcoming textural issues can further help in increasing consumer liking of yoghurt products, as texture is an important factor affecting acceptability.^42^ Knowing consumers perceptions and tasting responses can be used for a consumer‐oriented design process, keeping consumers as the focus rather than the product.^43^ Overall liking is affected by similar emotions. As the consumers are made more aware of the benefits of the plant‐based yoghurt product category, it may help to eliminate any perceptual bias, increasing the popularity of plant‐based yoghurts.

## CONCLUSION

The present study can help increase our understanding of the consumer attitudes and behaviours towards plant‐based yoghurt alternatives and how this differs with cultural background. If formulated into palatable form, plant‐based yoghurts have the potential to serve as an alternative to dairy. Many factors need to be kept in mind during the development of new plant‐based yoghurt alternatives, which include, achieving acceptable taste and texture, managing ingredient‐related expectations, delivering credible nutritional and health positioning, reducing negative expectations and emotions through appropriate product cues and communication, accounting for cultural familiarity and yoghurt usage contexts, aligning sustainability/diet positioning with target segments, and ensuring competitive price or value and willingness‐to‐pay. Future studies may extend the current work by exploring consumer psychology and the differences of cultural groups. Consumers also need to be made aware of the benefits of this new product category, which may increase the acceptability of plant‐based yoghurt alternatives. Consumer perceptions play an important part in yoghurt acceptability, even if the quality and texture are good. This needs to be addressed to improve better acceptance of these products. Future studies should compare perception‐based responses under informed conditions with blind tasting outcomes across a wider range of plant‐based yoghurt formulations and flavours, to quantify expectation effects and validate the generalizability of the present findings.

## CONFLICT OF INTEREST

The authors declare no conflict of interest, and the manuscript has been approved by all authors for publication.

## ETHICS STATEMENT

The study was approved by the Human Ethics Advisory Group (HEAG) of the Faculty of Veterinary and Agriculture Sciences (FVAS) at the University of Melbourne (Ethics ID 1853507.2) on 22 August 2019.

## Supporting information


**Table S1.** Demographics of the respondents in quantitative survey.
**Table S2**. Comparison of overall liking scores for dairy with plant‐based yoghurts for sub‐division of the demographic categories.
**Table S3**. List of emotion terms created with the focus group study (*n* = 32).

## Data Availability

The data that support the findings of this study are available from the corresponding author upon reasonable request.
