# Supplementary material for: Attitudes and behaviours of consumers towards plant‐based yoghurt alternatives: a cross‐cultural study
Source: J Sci Food Agric. 2026 May 19;106(11):6326–36. doi: 10.1002/jsfa.70638 (PMC13341057; doi:10.1002/jsfa.70638)
Supplement: Supplementary file 1 — Table S1. Demographics of the respondents in quantitative survey. Table S2. Comparison of overall liking scores for dairy with plant‐based yoghurts for sub‐division of the demographic categories. Table S3. List of emotion terms created with the focus group study (n = 32). [file JSFA-106-6326-s001.docx]

**Table S1.** Demographics of the respondents in quantitative survey

| Characteristic | Category | Number of consumers | Percentage participants |
| --- | --- | --- | --- |
| Gender | Female | 278 | 73% |
|  | Male | 105 | 27% |
|  |  |  |  |
| Age group | 17-24 years | 156 | 40% |
|  | 25-34 years | 94 | 24% |
|  | 35-44 years | 56 | 14% |
|  | >44 years | 84 | 22% |
|  |  |  |  |
| Ethnicity | Western | 260 | 60% |
|  | Asian | 173 | 40% |
|  |  |  |  |
| Work Status | Work Full-time | 130 | 31% |
|  | Student | 248 | 59% |
|  | Work Part-time | 45 | 11% |
|  |  |  |  |
| Food consumption habits | Not on a specific diet | 298 | 69% |
|  | Flexitarian | 57 | 13% |
|  | Vegan / vegetarian | 54 | 13% |
|  | Others | 22 | 5% |

**Table S2.** Comparison of overall liking scores for dairy with plant-based yoghurts for sub-division of the demographic categories.

| Interaction | Demographic | Sub-group | Dairy means | Plant means | P value |
| --- | --- | --- | --- | --- | --- |
| Product * Age group | Age Group (in years) | 17-24 | 6.60^a^ | 5.06^c^ | p <0.001 |
|  |  | 25-34 | 7.77^b^ | 5.17^c^ |  |
|  |  | 35-44 | 7.79^b^ | 5.52^c^ |  |
|  |  | >44 | 8.12^b^ | 5.12^c^ |  |
|  | | | | | |
| Product * Work Status | Work Status | Work Part-time | 8.04^a^ | 5.62^cd^ | p <0.001 |
|  |  | Work Full-time | 8.01^a^ | 5.32^cd^ |  |
|  |  | Student | 6.62^b^ | 5.04^d^ |  |
|  | | | | | |
| Product * Ethnicity | Ethnicity | Western | 7.61^a^ | 5.27^c^ | p = 0.001 |
|  |  | Asian | 6.62^b^ | 5.09^c^ |  |

*Means with different superscripts in each interaction indicate significant differences (p < 0.05) by the Fischer's LSD test. Superscripts denote statistically significant differences among means within each interaction block, including differences between dairy and plant-based products and across demographic sub-groups.*

**Table S3.** List of emotion terms created with the focus group study (n=32)

| **Sensory Emotion** | **Positive** | Cheerful, Luxury, Trusted, Uplifting, Dependable |
| --- | --- | --- |
|  | **Neutral** | Neutral, Guilt-free, Basic, Indifferent, Common |
|  | **Negative** | Nasty, Deceitful, Cheap, Artificial, Pretentious |
